# Dupilumab: Direct Cost and Clinical Evaluation in Patients with Atopic Dermatitis

**DOI:** 10.1155/2023/4592087

**Published:** 2023-02-15

**Authors:** Marco Ferrari, Matthew G. Donadu, Gabriele Biondi, Laura Saderi, Federica Sucato, Maria A. Montesu, Paola Ruggiu, Paola Merella, Carla Chessa, Angela Sias, Gabriella Carmelita, Vittorio Mazzarello, Giovanni Sotgiu, Satta Rosanna

**Affiliations:** ^1^Department of Biomedical Sciences, University of Sassari, Sassari, Italy; ^2^Hospital Pharmacy, Sassari University Hospital, Sassari, Italy; ^3^Department of Clinical and Experimental Medicine, Dermatology Unit, University of Sassari, Sassari, Italy; ^4^Clinical Epidemiology and Medical Statistics Unit, Department of Medical, Surgical and Experimental Sciences, University of Sassari, Sassari, Italy

## Abstract

Health care spending in Italy is high and continues to increase; assessing the long-term health and economic outcomes of new therapies is essential. Atopic dermatitis (AD) is a chronic, pruritic, immune-mediated inflammatory dermatosis, a clinical condition that significantly affects patients' quality of life at a high cost and requires continuous care. This retrospective study aimed to assess the direct cost and adverse drug reactions (ADRs) of Dupilumab and patients' clinical outcomes. All AD patients treated with Dupilumab at the Sassari University Hospital, Italy, between January 2019 and December 2021 were included. Eczema Area Severity Index, Dermatology Life Quality Index, and Itch Numeric Rating Scale scores were measured. ADRs and drug expenses were analyzed. A statistically significant posttreatment improvement was observed for all the indices measured: EASI (*P* < 0.0001), DLQI (*P* < 0.0001), NRS (*P* < 0.0001). The total expenditure for Dupilumab, in the observed period, amounted to € 589.748,66 for 1358 doses, and a positive correlation was shown between annual expenditure and delta percentage of variation pre- and posttreatment for the clinical parameters evaluated.

## 1. Introduction

Atopic dermatitis (AD) is a chronic, recurrent inflammatory skin disease with a complex pathogenesis involving genetic susceptibility, immunological dysfunction, the epidermal barrier, and environmental factors. AD is one of the most studied dermatological diseases in recent years, especially with the recent progress with the use of biological therapies, of which the first approved drug for moderate-to-severe AD is Dupilumab. Dupilumab (Dupixent®, Regeneron) is a fully human monoclonal antibody that targets the interleukin (IL)-4 receptor-*α*. This binding to IL-4*α* inhibits signaling of the Th2 cytokines IL-4 and IL-13 that contribute to the pathogenesis of AD [1]. A recent systematic review was performed, which included more than 7,000 articles with data from all continents on children and adults. Each year, up to 17.1% of adults and 22.6% of children were diagnosed with AD, with as many as 9.6% of new cases of AD in children [2]. In Italy, the lifetime prevalence of AD was estimated between 15 and 17% in schoolchildren and between 8 and 13% in adolescents [3, 4]. The purpose of this study was to assess the drug expenditure related to Dupilumab treatment for AD between January 2019 and December 2021 at the Sassari University Hospital, Northwest Sardinia, Italy. Furthermore, an epidemiological analysis of AD patients treated with Dupilumab has been analyzed, considering clinical outcome, therapy adherence, and AD scores.

## 2. Materials and Methods

All 57 AD patients (34 men and 23 women; mean age, 31 years) treated with Dupilumab at the Dermatology Clinic, Azienda Ospedaliera Universitaria, Sassari, Italy, between January 2019 and December 2021 were included in this retrospective study. During the observation period, all the patients had at least 4 months of treatment with Dupilumab up to 3 years, in order to assess the clinical outcome. Our unit has a catchment population of approximately 335,000 living in an area of 4300 square kilometers (Sassari province). Ethical approval was waived by the local Ethics Committee of the Azienda Ospedaliero-Universitaria di Sassari given the retrospective nature of the survey, which was conducted in complete agreement with the principles of the Declaration of Helsinki. Each participant received detailed information and provided informed consent. Patients received subcutaneous Dupilumab 300 mg with an initial loading dose of 600 mg at time zero, followed by 300 mg after two weeks, continuing with 300 mg every other week. One pediatric patient was administered with the 200 mg dose on a similar schedule. At each visit and throughout the study period, the dermatologist performed a subjective clinical assessment of AD using the Eczema Area Severity Index (EASI). Each patient, through an Itch Numeric Rating Scale (NRS), compiled a diary that recorded the overall severity of the skin condition, the severity of itching, and sleep disturbances. A questionnaire was placed at each clinical control to evaluate the Dermatology Life Quality Index (DLQI).

### 2.1. Data Analysis

Data regarding the treatment and regimen were extracted from the dermatology clinic records, web-based monitoring records by the Italian Medicines Agency (Agenzia Italiana del Farmaco-AIFA), and data flows included in the New Health Information System (Nuovo Sistema Informativo Sanitario-NSIS). Data on safety were evaluated as the number and severity of suspected adverse reactions to drugs (adverse drug reactions, or ADRs) observed during the analysis of medical records. The costs of human monoclonal antibodies under analysis were extracted by the IT system of the Sardinian Region, adopted by all healthcare facilities in Sardinia.

### 2.2. Statistical Analysis

Demographic and clinical variables were collected in an electronic database. Qualitative data were summarised with absolute and relative frequencies (percentages), whereas quantitative data were described with medians and interquartile ranges (IQRs) based on the nonparametric distribution of the variables. The Wilcoxon signed-rank test was used to compare pre- and posttreatment clinical outcomes. Spearman's correlation was calculated to assess the relationship between the percentage variation in clinical indices before and after treatment and the annual expenditure for Dupilumab. A two-tailed *p* value <0.05 was considered statistically significant. STATA software (StataCorp, Texas, USA), version 17, was adopted for all statistical analyses.

## 3. Results

The number of patients with moderate-to-severe AD treated with Dupilumab was 57. The total treatment cost during the analyzed period amounted to € 589.748,66 for 1358 doses. 798 doses for males and 564 for females corresponding, respectively, to 343.774,45 euros and 345.974,19 €. In 2019, the total expenditure related to Dupilumab treatment amounted to 24.710,37 € for 54 doses; 34 doses for males and 20 doses for females corresponding, respectively, to 15.558,38 € and 9.151,99 €. In 2020 the total expenditure amounted to 258.976,2 € for 566 doses; 310 doses for males; and 256 doses for females corresponding, respectively, to 141.841,73 € and 117.134,45 €. In 2021, the total expenditure amounted to 306.062,09 € for 738 doses: 450 doses for males and 288 for females corresponding, respectively, to 186.374,34 € and 119.687,75 € (Table 1). The area most frequently affected was face (84.2%) followed by hands (73.7%) and genital areas (49.1%). Previous treatments included topical steroids (91.2%), topical immunomodulators (45.6%), systemic corticosteroids (45.6%), and cyclosporine (43.9%). Among the comorbidities, asthma was present in 54.4% of patients followed by allergic rhinitis (26.3%). Median EASI, DLQI, and NRS were, respectively, 31, 10, and 10 at baseline reduced to 5, 3, and 2, respectively, at the end of the treatment (Table 2). No major ADRs were reported.

## 4. Discussion

**Figure 1:**
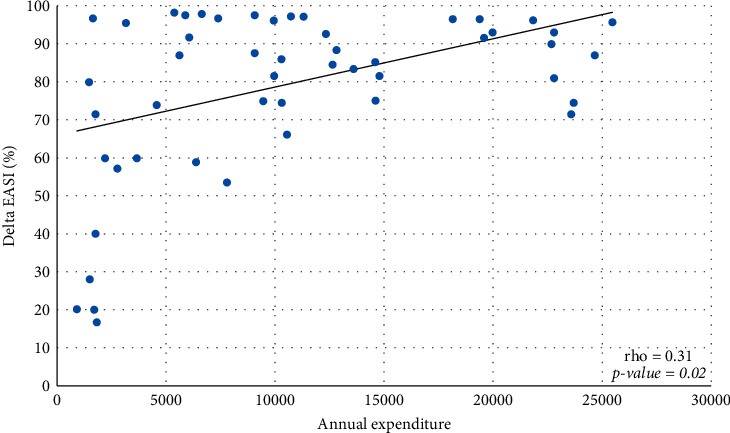
Relationship between annual expenditure for Dupilumab and delta EASI percentage.

**Figure 2:**
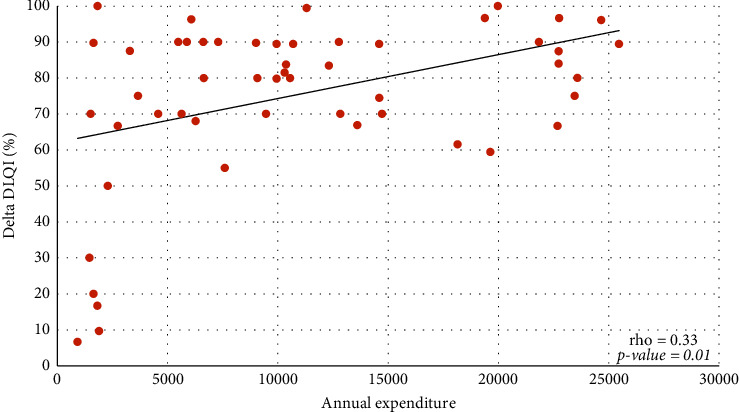
Relationship between annual expenditure for Dupilumab and delta DLQI percentage.

**Figure 3:**
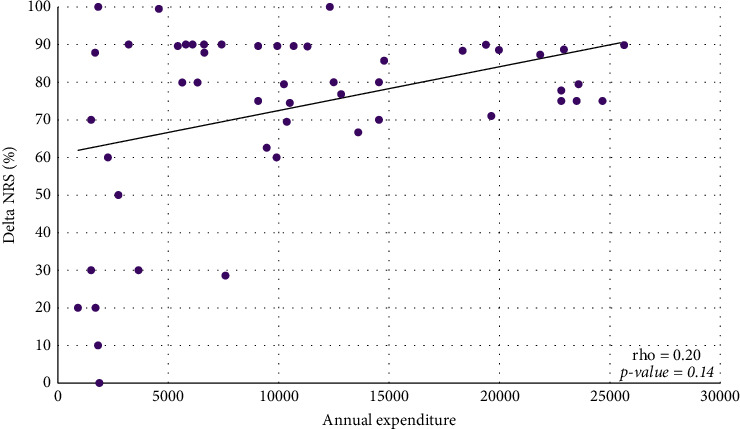
Relationship between annual expenditure for Dupilumab and delta NRS percentage.

Most physicians are involved in the care of patients with AD, a disease which, depending on the severity of the clinical form, can significantly influence the quality of life of the affected individuals. Itching often disrupts sleep, leading to daytime drowsiness and irritability, which can lead to psychological stress and impaired performance at school and work. Most patients with atopic dermatitis use bland moisturizers and emollients for treatment, along with meticulous and often difficult or lengthy skin care routines [5–7]. The main therapeutic goals in the management of AD are the control of symptoms and the prevention of skin inflammation. Topical therapies have a considerable cost for the patient and the national health system (SSN). In past years, most of these patients with medium-severe forms of AD were hospitalized and treated with intravenous therapy. In recent years, the discovery of therapies with monoclonal antibodies has led to a significant change in the management of these patients. Biologic treatments are more effective than conventional therapies and ensure a sustained response with an adequate risk-benefit profile, due to their targeted effect [8]. Indeed, patient management with the use of novel therapy such as the Dupilumab changed and improved patients' quality of life. Dupilumab is an IgG4 human monoclonal antibody (mAb) that binds IL-4Ra [9, 10] with subsequent inhibition of IL-4R signaling induced by both IL-4 and IL-13, and down-regulation of TH2 inflammation in a variety of allergic disorders, including atopic dermatitis, asthma, and possibly other allergic diseases. Dupilumab is the first biologic treatment that effectively addresses the pathophysiology of TH2 allergic diseases, combining therapeutic efficacy with a low incidence of adverse events. It can target fundamental mechanisms in TH2 cell inflammatory diseases by blocking TH2 cell differentiation, IgE production by B cells, alternative macrophage activation, and other hallmarks of allergic inflammatory diseases [11]. Our work shows a retrospective analysis of patients with AD receiving Dupilumab analysing direct costs and clinical outcomes. Value-based frameworks requiring estimation of overall cost-effectiveness have been recommended to help clinicians and payers compare the value of medications and other health technologies across multiple therapeutic areas on a common scale [12]. A recent health economic model was developed to evaluate from the US payer perspective the long-term costs and benefits of Dupilumab treatment administered every other week. Dupilumab, compared with supportive care, is cost-effective for treating moderate-to-severe AD in US adults at an annual maintenance price ranging from $29,000–$40,000 to $100,000–$150,000 per quality-adjusted life-year decision thresholds [13]. These costs can be considered slightly lower compared with our data of the average annual cost for the analyzed period for Dupilumab being 196.582 €. In Italy, the reimbursement criteria are envisaged for treatment with Dupilumab by the SSN in adult patients with atopic dermatitis for whom treatment with cyclosporine is contraindicated, ineffective, or not tolerated, and EASI score ≥24. Reimbursement by the SSN for Dupilumab occurs in children and adolescents aged 6 to 17 years with severe atopic dermatitis eligible for systemic therapy, who have an EASI score ≥24 or one of the following criteria: localization in areas visible and/or sensitive; evaluation of pruritus with NRS ≥7 scales; and assessment of the quality of life with a DLQI index ≥10. A positive correlation was shown between annual expenditure for Dupilumab and delta percentage of variation pre- and posttreatment for the clinical parameters evaluated (Table 3). The median EASI reduced from 31 at baseline to 5 at the end of the treatment with a Delta EASI percentage of 86.8 (Figure 1) and a statistically significant posttreatment improvement (*P* < 0.0001). The median DLQI reduced from 10 at baseline to 3 at the end of the treatment, with a Delta DLQI percentage of 80.0 (Figure 2) and a statistically significant posttreatment improvement (*P* < 0.0001). The median NRS reduced from 10 at baseline to 2 at the end of the treatment with a Delta NRS percentage of 80.0 (Figure 3), with a statistically significant posttreatment improvement (*P* < 0.0001). These results required a median annual cost for patient of 9073 €. It is difficult to estimate the real annual cost for patient under systemic or topical treatment as the following variables need to be taken into consideration. In recent years, patients with moderate-to-severe AD, due to the clinical manifestations, were frequently hospitalized for long periods to undergo systemic treatments, often intravenously. Patient management, therefore, involved long periods of hospitalization. To date, this type of hospitalization is no longer performed, and the management of patients treated with monoclonal antibodies takes place through outpatient check-ups resulting in considerable savings for the SSN. Additional costs include screening, monitoring, and prescription of standard systemic treatments such as corticosteroids, cyclosporine, and azathioprine. Dupilumab treatment also reduces the use of antihistaminic intake and supportive topical treatment and emollients. As evidenced by our analysis, the initiation of therapy with monoclonal antibodies has significantly decreased the use of other treatments with benefit for the patient and the SSN. The significant improvement due to Dupilumab treatment compared with the other standard treatment reasonably reduces morbidity with related costs [13]. Among the most represented comorbidities were asthma, allergic rhinitis, and/or conjunctivitis as reported in literature. Although the satisfactory therapeutic outcome of the Dupilumab treatment was reported, a small number of paper analysing the cost evaluation compared to the clinical outcome were reported in the literature [14]. Furthermore, the safety of the treatment is reported in a few works; our retrospective analysis also highlights the safety of the treatment, and no ADRs have been registered, compared with ADRs and side effects of standard systemic treatments that can increase costs for SSN and patients.

## 5. Conclusion

The favorable benefit-risk-cost profile in this study supports the long-term role of Dupilumab treatment for patients with moderate-to-severe AD. Although the Dupilumab treatment direct cost might appear higher than standard treatments, when taking in consideration the overall expenditure sustained by the SSN and patients, the improvement of the quality of life, the reduced use of standard topical and systemic treatments, the reduction of screening and monitoring patients, and the reduction of morbidity, it is reasonable to state that the overall cost-effectiveness might be advantageous for Dupilumab. The introduction of novel therapies has a cost that health systems will have to foresee in the coming years, considering the overall clinical benefits and the reduction in the number of hospitalizations, especially in a pandemic era.

## Figures and Tables

**Table 1:** Annual dispensing number and cost of Dupilumab in moderate-to-severe atopic dermatitis in the period January 2019 and December 2021.

Gender	2019		2020		2021	
	Annual expenditure	Drug dispensing number	Annual expenditure	Drug dispensing number	Annual expenditure	Drug dispensing number
Females	9.151,99 €	20	117.134,45 €	256	119.687,75 €	288
Males	15.558,38 €	34	141.841,73 €	310	186.374,34 €	450
Total	24.710,37 €	54	258.976,2 €	566	306.062,09 €	738

**Table 2:** Median outcome and descriptive analysis of patients with moderate-to-severe atopic dermatitis.

Variables	
Males, *n* (%)	34/57 (59.7)
Median (IQR) age, years	31 (24–47)

*Treatment outcome*	
Median (IQR) annual expenditure/patient (euro)	9073 (3661–14600)
Median (IQR) drug dispensing number	22 (8–34)
Median (IQR) EASI at baseline	31 (25–40)
Median (IQR) EASI at the end of treatment	5 (1–10)
Median (IQR) delta EASI (%)	86.8 (75–96)
Median (IQR) DLQI at baseline	10 (10–25)
Median (IQR) DLQI at the end of treatment	3 (1–5)
Median (IQR) delta DLQI (%)	80 (70–90)
Median (IQR) NRS at baseline	10 (8–10)
Median (IQR) NRS at the end of treatment	2 (1–3)
Median (IQR) delta NRS (%)	80 (70–90)
Monotherapy, *n* (%)	44/56 (78.6)

*Comorbidities*	
None, *n* (%)	17/57 (29.8)
Allergic asthma, *n* (%)	31/57 (54.4)
Allergic rhinitis and/or conjunctivitis, *n* (%)	15/57 (26.3)
Bacterial skin infections, *n* (%)	4/57 (7.0)
Recurrent herpetic infections, *n* (%)	2/57 (3.5)
Neuropsychiatric diseases, *n* (%)	0/57 (0.0)

*Affected areas*	
Face/neck, *n* (%)	48/57 (84.2)
Body, *n* (%)	3/57 (5.3)
Genitals, *n* (%)	28/57 (49.1)
Hands, *n* (%)	42/57 (73.7)

*Previous treatment*	
Topical corticosteroids, *n* (%)	52/57 (91.2)
Topical immunomodulators (e.g., tacrolimus, pimecrolimus), *n* (%)	26/57 (45.6)
Systemic corticosteroids, *n* (%)	26/57 (45.6)
Cyclosporine, *n* (%)	25/57 (43.9)
Methotrexate, *n* (%)	1/57 (1.8)
Azathioprine, *n* (%)	0/57 (0.0)
NB-UVB phototherapy, *n* (%)	1/57 (1.8)
UVA1 phototherapy, *n* (%)	0/57 (0.09)
PUVA phototherapy, *n* (%)	1/57 (1.8)

**Table 3:** Comparison of clinical outcomes pre- and posttherapy with Dupilumab.

	At baseline	At the end of observation period	*p* value
Median (IQR) EASI at baseline	31 (25–40)	5 (1–10)	<0.0001
Median (IQR) DLQI at baseline	10 (10–25)	3 (1–5)	<0.0001
Median (IQR) NRS at baseline	10 (8–10)	2 (1–3)	<0.0001

## Data Availability

The datasets generated during and/or analyzed during the current study are available from the corresponding author upon reasonable request.
